# Relevance of Prescribing Serum Immunofixation Electrophoresis in the Diagnosis of Monoclonal Gammopathies

**DOI:** 10.7759/cureus.86339

**Published:** 2025-06-19

**Authors:** Hajar Fadili, Hamza Ouazzani, Ikrame Rhaleb, Ines Bendi, Malak Snoussi, Abdelhamid Zrara, Youssef Bamou, Jalila El Bakkouri

**Affiliations:** 1 Biology, Hôpital Universitaire International Cheikh Khalifa Ibn Zaid, Université Mohammed VI des Sciences et de la Santé (UM6SS), Casablanca, MAR

**Keywords:** diagnosis of multiple myeloma, monoclonal band, monoclonal gammopathy, serum immunofixation electrophoresis, serum protein electrophoresis

## Abstract

Introduction

Immunofixation electrophoresis (IFE) is an immunoprecipitation technique that combines the electrophoretic separation of serum proteins with the identification of immunoglobulin heavy chains (IgG, IgA, IgM) and light chains (κ or λ) using targeted antisera. It is typically used as a complement to serum protein electrophoresis (SPE) when abnormal profiles, such as a monoclonal peak, are detected. IFE is a reference method for confirmation and phenotyping in the diagnosis of monoclonal gammopathies (MGs). This study aims to evaluate the diagnostic yield of IFE compared to SPE in patients with suspected MGs. It also seeks to assess whether current prescription practices for IFE, when used as a complement to SPE, align with clinical guidelines to minimize unnecessary testing.

Materials and methods

This retrospective study was conducted at the laboratory of Hôpital Universitaire International Cheikh Khalifa Ibn Zaid, Casablanca, Morocco, from January 2023 to January 2025. We collected 200 serum samples from patients with clinical suspicion of MG who underwent IFE performed on agarose gel using the HYDRASYS system (Sebia, Lisses, France).

Results

Among the 200 patients included in the study, 103 (51.5%) were men and 97 (48.5%) were women, resulting in a sex ratio of 1.06. The average age was 63 years. IFE was performed on all 200 samples and identified monoclonal bands in all 79 patients with MGs (100%), including the 55 cases (69.6%) that had monoclonal peaks detected by SPE. Thus, our study achieved a 100% detection rate of MGs by IFE, whereas SPE identified only 69.6%.

Discussion

IFE consistently demonstrates superior sensitivity to SPE. These data highlight the difficulty of interpreting electrophoretic profiles and the need to decide whether to follow up with IFE or simply monitor the patient. Although a normal SPE does not rule out the diagnosis of multiple myeloma (MM), emphasizing the need for IFE in cases of strong clinical suspicion, our series reveals that for a very large proportion of patients, SPE, IFE, and serum free light chain (FLC) assays were simultaneously requested by the clinician and all turned out negative. This underscores the importance of better prioritizing tests to avoid excessive and potentially unnecessary prescriptions for additional analyses. In all cases, direct communication between the biologist and the clinician is recommended.

Conclusion

While SPE is a useful and economical initial screening tool, IFE is essential for an accurate diagnosis of related monoclonal gammopathies due to its superior sensitivity and specificity. IFE, combined with complementary analyses such as serum FLC assays, currently constitutes the reference method for definitive identification.

## Introduction

Serum immunofixation electrophoresis (IFE) is an immunoprecipitation laboratory technique that combines electrophoretic separation of serum proteins with the specific identification of immunoglobulin heavy (IgG, IgA, IgM) and light chains (κ or λ) using targeted antisera. IFE is typically used after serum protein electrophoresis (SPE), which serves as an initial screening method to detect abnormal protein patterns such as monoclonal protein spikes. Thus, IFE and SPE are complementary, with SPE screening for abnormalities and IFE serving as the gold standard for confirmation and characterization in the diagnostic evaluation of monoclonal gammopathies (MGs) [1]. This study aims to evaluate the diagnostic yield of IFE compared to SPE in patients with suspected MGs. It also seeks to assess whether current prescription practices for IFE, when used as a complement to SPE, align with clinical guidelines to minimize unnecessary testing.

## Materials and methods

This retrospective study was carried out at the medical laboratory of Hôpital Universitaire International Cheikh Khalifa Ibn Zaid, affiliated with Université Mohammed VI des Sciences et de la Santé (UM6SS) in Casablanca, Morocco. Data was collected from January 2023 to January 2025.

The study included 200 blood samples from patients with a clinical suspicion of MG, which was operationally defined as the presence of at least one documented trigger on the test request: unexplained anaemia, renal impairment, hypercalcaemia, lytic bone lesions or persistent bone pain, hyperproteinaemia, markedly elevated inflammatory markers without infection, unexplained peripheral neuropathy, significant proteinuria, recurrent bacterial infections. To ensure data integrity, we excluded patients with a previous diagnosis of myasthenia gravis, those who did not initially receive a SPE, those with insufficient sample volume or whose hemolysis could compromise IFE or SPE results, and those with incomplete clinical data required for accurate interpretation of laboratory results.

**Figure 1:**
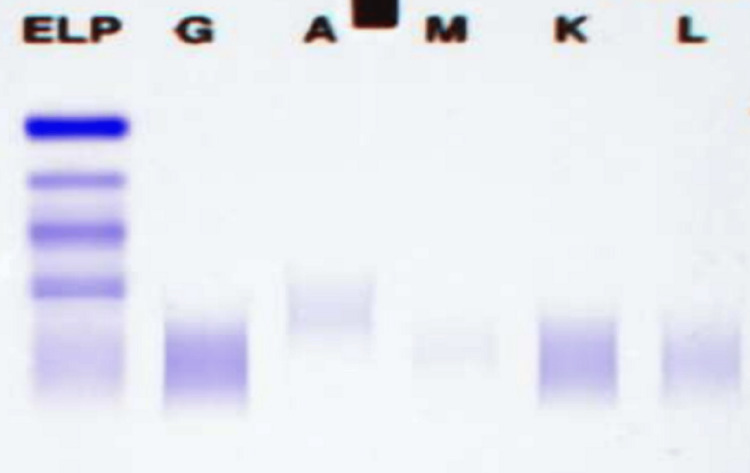
Immunofixation electrophoresis showing no monoclonal band.

For all these patients, IFE was performed using the HYDRASYS 2 scan system (Sebia, Lisses, France) on agarose gel. The IFE technique, which allowed for the identification of the specific type of heavy chain (IgG, IgM, or IgA) and light chain (κ or λ) as visually represented in Figure 1, begins with the application of the fresh serum sample, ensuring that antigen excess (the "prozone effect") is avoided by using diluted samples. This initial step is followed by the electrophoretic migration of proteins. Next, specific antisera are then applied: 40 µL for the electrophoresis lane and 25 µL for the IgA, IgG, IgM, κ, or λ (A, G, M, K, L) lanes. Immune complex precipitation then takes place, followed by gel aspiration (pumping) and drying. Finally, the gel is washed and stained. In accordance with the manufacturer's recommendations, serum is diluted using the diluent supplied in the kit, which contains bromophenol blue. For IgG, a 1/6 dilution is generally used, as these are the predominant immunoglobulins in serum. For the other tracks (IgA, IgM, κ, λ), a 1/3 dilution is applied. These dilutions are adjusted if necessary for hyper- or hypogammaglobulinemia, to ensure accurate results. The analysis is performed entirely with the HYDRASYS automated system. The complete analysis kit includes buffered wicks, hydragel gel, applicator, acid violet dye, diluent for patient samples, as well as three types of paper and washing solution.

The 200 patients initially underwent SPE, performed on a Sebia Capillarys system producing digital densitometric tracings. The raw data are numerical absorbance values that feed directly into the area-under-curve (AUC) calculations.

Following data collection and initial screening, statistical analysis was performed using JAMOVI software (Version 2.3.21) (https://www.jamovi.org) to perform descriptive statistics (frequencies and percentages) to characterize the distribution of different patterns among the study population, and to generate tables, aiding in the clear presentation of our results.

## Results

**Figure 2:**
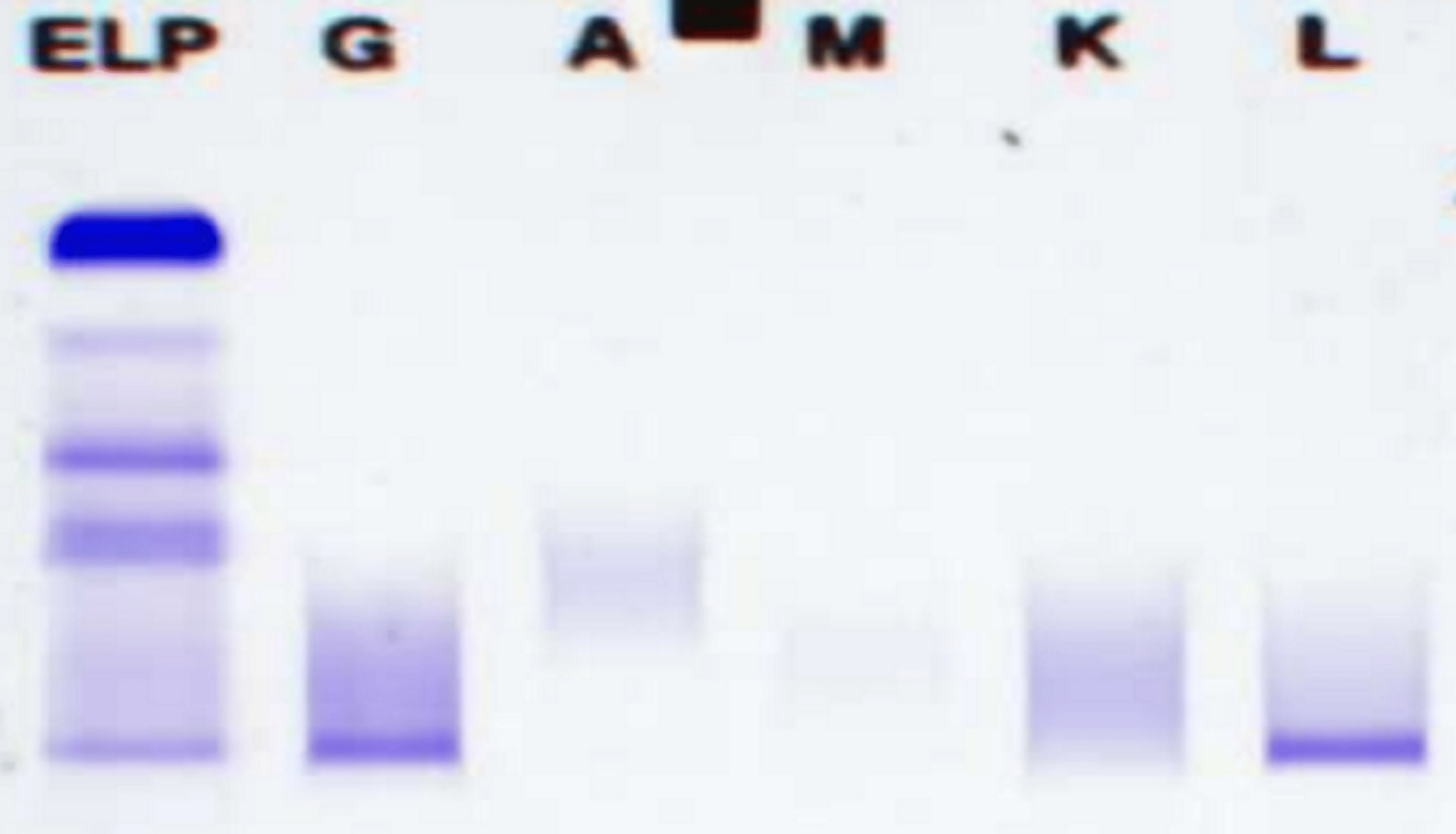
Immunofixation electrophoresis showing a positive serum IgG-λ

Of the 200 patients included in the study, 103 (51.5%) were male and 97 (48.5%) were female, yielding a male-to-female ratio of 1.06. The mean age was 63 years, with extremes ranging from 42 to 80 years. IFE results revealed monoclonal bands (M-bands) as narrow, dense, concentrated, and clearly defined precipitation bands, positioned horizontally aligned with the M-band on the reference lane, as shown in Figure 2. Table 1 shows the distribution of positivity in IFE.

**Table 1:** Distribution of positivity in IFE. IFE: immunofixation electrophoresis

IFE positivity	Number of cases (n=200)	Percentage
Monoclonal band	79	39.5%
No Monoclonal band	121	60.5%

Table 2 shows the distribution of heavy and light chain isotypes among the 79 cases of monoclonal gammopathy.

**Table 2:** Distribution of patterns in IFE. IFE: immunofixation electrophoresis

Immunophenotyping	Type κ, n (%)	Type λ, n (%)	Total (n=79), n (%)
IgG	30 (37.97%)	19 (24.05%)	49 (62.02%)
IgA	8 (10.13%)	3 (3.80%)	11 (13.92%)
IgM	2 (2.53%)	3 (3.80%)	5 (6.33%)
Light Chains	8 (10.12%)	6 (7.59%)	14 (17.72%)

All 200 patients underwent an initial SPE. Table 3 summarizes the distribution of IFE positivity, correlated with the SPE profile observations, which detected a monoclonal peak in 55 cases. The remaining 145 cases showed hypogammaglobulinemia, restriction of heterogeneity in gamma globulins, a normal profile, or non-specific profiles. IFE successfully detected MG in all cases of monoclonal peaks objectively identified by SPE.

**Table 3:** Distribution of IFE positivity, correlated with observed SPE profiles and immunophenotyping results IFE: immunofixation electrophoresis; SPE: serum protein electrophoresis

SPE pattern	Number of patients (n=200)	Rate of monoclonal band detection at the IFE, n (%)	Immunophenotyping
Monoclonal protein migrating in the gamma region	50	50/50 (100%)	28 IgG κ - 15 IgG λ - 3 IgA κ - 2 IgA λ - 1 IgM κ - 1 IgM λ
Monoclonal protein migrating in the beta-2 region	5	5/5 (100%)	2 IgA κ - 2 light chains λ - 1 IgG κ
Hypogammaglobulinemia	34	12/34 (35.2%)	8 light chains κ - 4 light chains λ
Heterogeneity restrictions	28	10/28 (35.7%)	3 IgG λ - 2 IgM λ - 2 IgA κ - 1 IgA λ - 1 IgM κ - 1 IgG κ
Increased beta-2 fraction	5	1/5 (20%)	IgA κ
Splitting of the beta-2 fraction	1	1/1 (100%)	IgG λ
Non-specific findings (Polyclonal Gammopathy, Beta-gamma bridge, Acute Inflammation, Hypoalbuminemia, and Normal Profiles)	77	0/77 (0%)	- - -

IFE detected MGs in 12 out of 34 cases of hypogammaglobulinemia. The remaining hypogammaglobulinemia cases primarily consisted of patients with exudative enteropathy, common variable immunodeficiency, or those on immunosuppressants. Similarly, MG was identified in 10 out of 28 cases of restricted gamma heterogeneity. The remaining cases of restriction were composed of patients with inflammatory conditions, such as autoimmune diseases or viral/bacterial infections.

IFE did not detect any bands in cases with non-specific profiles such as polyclonal hypergammaglobulinemia, beta-gamma bridging, inflammatory profiles, hypoalbuminemia, and normal profiles. Thus, our study achieved 100% detection of MGs by IFE, whereas SPE only identified 69%.

## Discussion

In multiple myeloma (MM), malignant plasma cells synthesize monoclonal antibodies, leading to an increased level of monoclonal protein in the serum [2]. In our study, the most common M-band was IgG κ, followed by IgG λ, consistent with findings reported by Uddin et al. [3] and Tarek et al. [4]. 

IFE consistently demonstrates superior sensitivity to SPE, particularly for detecting low-concentration monoclonal proteins and early relapsed disease. While our study achieved 100% detection of MGs using IFE, SPE identified only 69.6%. This discrepancy is consistent with lower SPE detection rates reported in other studies; Uddin et al. found 44.3% M-protein positivity [3], Chopra et al. found 24.4% [5], and Tarek et al. found 6.3% [4]. The higher detection rates in our study and in Uddin et al.'s [3] series likely reflect the selection of clinically high-suspicion MG cases, whereas lower rates in other studies may result from random sample selection. Notably, the absence of a narrow peak on SPE does not exclude myeloma, including light chain (10% of myelomas) and non-secretory types [6]. Furthermore, IFE's ability to detect relapsed MM earlier underscores its importance in long-term patient monitoring [7-9].

Although SPE remains a valuable screening tool, IFE confirmation is crucial, especially for light chain myelomas and peaks in atypical regions such as α2 or β, instead of the common γ-region [3-10]. Also, SPE can yield false negatives when low-concentration paraproteins co-migrate with normal protein fractions, as seen in light chain disorders. This was observed in our patient who had an elevation of the beta-2 fraction, where complement co-migrated with an IgA κ band. Thus, IFE reveals missed diagnoses by directly identifying immunoglobulin and clonality [11,12]. 

IFE detected no M-bands in 12 of 34 (35.29%) hypogammaglobulinemia cases. Similarly, no M-bands were identified in 10 of 28 (35.71%) restriction cases. These data highlight the difficulty in interpreting electrophoretic profiles and deciding whether or not to supplement with IFE or simply monitor. The French Haute Autorité de Santé (HAS) in its recommendations also advises against systematically performing an IFE in cases of restriction heterogeneity. In all cases, direct communication between the biologist and the clinician is recommended [13].

Out of 200 patients, 77 (38.5%) had negative IFEs and presented with normal SPE or non-specific findings (polyclonal gammopathy, beta-gamma bridging, acute inflammation, hypoalbuminemia). Although a normal SPE does not rule out the diagnosis of multiple myeloma, thus highlighting the need to supplement with IFE and serum FLC assay in cases of high clinical suspicion [14,15], our series reveals a common practice where IFE and FLC assays are prescribed concomitantly with SPE. Indeed, a large proportion of these patients presented with normal SPE, yet IFE and serum FLC assays were requested simultaneously by the clinician and proved negative. This highlights the importance of better prioritizing tests to avoid excessive and potentially unnecessary prescription of additional analyses.

Our study has certain inherent design limitations. Firstly, the deliberate selection of patients with a high clinical suspicion of MGs likely led to higher detection rates than would be observed in a general or screening population. This approach could potentially overestimate detection sensitivity compared with studies using unselected samples. Secondly, this study was deliberately limited to the evaluation of laboratory utilization. Our primary objective was to measure the incremental diagnostic yield and avoidable cost of simultaneous SPE and IFE prescriptions, questions that can only be answered with laboratory data. The study is still aimed at improving diagnostic practices within laboratories and guiding clinicians in the appropriate use of tests, rather than assessing the long-term clinical benefits of an IFE diagnosis itself.

The strengths of our article lie in its focused review of when and why clinicians should order IFE, its fully detailed and reproducible IFE protocol, and its clear, guideline-based recommendations that incorporate health-economic considerations. High-quality figures and tables, along with a transparent limitations section, further enhance its utility for current practice and future refinement of diagnostic algorithms.

## Conclusions

IFE is the gold standard for the diagnosis of MGs, while SPE remains a useful and cost-effective initial screening test. In our laboratory, the direct reagent and labor cost of IFE was approximately six-fold higher than that of SPE. Our study investigated the frequency and consequences of the simultaneous prescription of IFE and SPE, in order to evaluate its impact in terms of cost and diagnostic efficiency. This approach highlights the need to rationalize complementary tests, prioritizing a rigorous interpretation of SPE to guide subsequent prescriptions.
